# Impact of SARS-CoV-2-specific memory B cells on the immune response after mRNA-based Comirnaty vaccine in seronegative health care workers

**DOI:** 10.3389/fmicb.2022.1002748

**Published:** 2022-09-23

**Authors:** Alejandro Vallejo, Pilar Vizcarra, Adrián Martín-Hondarza, Sandra Gómez-Maldonado, Johannes Haemmerle, Héctor Velasco, José L. Casado

**Affiliations:** ^1^Laboratory of Immunovirology, Department of Infectious Diseases, Ramon y Cajal Institute for Health Investigation (IRyCIS), University Hospital Ramón y Cajal, Madrid, Spain; ^2^Department of Infectious Diseases, Ramon y Cajal Institute for Health Investigation (IRyCIS), University Hospital Ramón y Cajal, Madrid, Spain; ^3^Department of Prevention of Occupational Risks, University Hospital Ramón y Cajal, Madrid, Spain

**Keywords:** SARS-CoV-2, vaccine, specific memory B cells, immune response, T cell response

## Abstract

**Purpose:**

To analyze the impact of SARS-COV-2-specific memory B cells (MBC) on the immune response after two doses of mRNA-based Comirnaty COVID-19 vaccine in seronegative health care workers. This study is seeking a rationale for boosting vaccines.

**Methods:**

Longitudinal study including 31 seronegative health care workers with undetectable specific MBCs (IgG^−^MBC^−^ group), 24 seronegative with detectable specific MBCs (IgG^−^MBC^+^ group), and 24 seropositive with detectable specific MBCs (IgG^+^MBC^+^ group). The level of antibodies that inhibit ACE2-RBD interaction, and anti-Spike IgG, IgA, and IgM antibodies was quantified by ELISA. In addition, specific memory B and T cells were quantified by flow cytometry.

**Results:**

The level of specific MBCs, and isotypes, in the IgG^−^MBC^−^ group was lower compared to that found in IgG^−^MBC^+^ (*p* = 0.0001) and IgG^+^MBC^+^ (*p* < 0.0001) groups, respectively. ACE2-RBD neutralizing antibodies and anti-S IgG antibodies were at lower levels in the IgG^−^MBC^−^group after the vaccine. Specific MBCs directly correlated with specific CD4^+^ T cells (although not significant, *p* = 0.065), while no correlation was found with specific CD8^+^ T cells (*p* = 0.156) after the vaccine. In parallel, ACE2-RBD neutralizing antibodies only positively correlated with specific CD4^+^ T cells (*p* = 0.034).

**Conclusion:**

IgG^−^MBC^−^ individuals showed the worst humoral and cellular responses, both in frequency and magnitude, after vaccination. Individuals whose antibodies wane and become undetectable after a given period of time post vaccination and show no specific MBCs are less protected and hence are good candidates for boosting vaccine. On the other hand, seronegative individuals with specific MBC showed faster and higher responses compared to the IgG^−^MBC^−^ group.

## Introduction

The introduction of wide vaccination has changed the SARS-CoV-2 infection spread and reduced COVID-19 deaths worldwide. Also, vaccination has allowed the reduction of the restrictions that characterized the pre-vaccine pandemic. As of May 2022, more than 11 billion vaccine doses have been administered worldwide (World Health Organization, 2022). However, the duration of protection induced by vaccination is still under investigation.

Several studies have shown that neutralizing antibody titers against the SARS-CoV-2 spike protein persisted for at least 5–8 months after infection (Abayasingam et al., 2021; Reyes et al., 2021) and declined slowly thereafter. Even at low titers, neutralizing antibodies have been associated with protection from severe disease (Dan et al., 2021; Ponticelli et al., 2021; Tsatsakis et al., 2021). However, immune response to SARS-CoV-2 includes specific memory T and B cells that can last up to 8 months after infection (Abayasingam et al., 2021; Dan et al., 2021; Giannotta and Giannotta, 2021). Many studies have focused on the analysis of neutralizing and binding antibody responses, while fewer have focused on memory T cell responses, and limited studies have been reported on specific memory B cells (MBC), two important factors that account for complete immune protection.

Hence, the absence of specific antibodies is not necessarily associated with the absence of specific memory T or B cells that could be reactivated promptly after eventual vaccine breakthrough. This is important for vaccination strategies in terms of boosting vaccine in vulnerable groups and protect them against the emergence of new variants of concern (Giannotta and Giannotta, 2021). Specific MBCs are maintained or even increased for at least 6 months to 1 year following SARS-CoV-2 infection (Hartley et al., 2020; Sokal et al., 2021; Turner et al., 2021; Wang et al., 2021).

Here, we analyzed the impact of specific MBCs on the immune response before vaccination and their association with other immunological parameters after two doses of mRNA-based Comirnaty (BNT162b2, Pfizer-BioNTech, United States) COVID-19 vaccine in seronegative individuals in a well-characterized cohort of health care workers followed since the beginning of the pandemic (March 2020).

## Materials and methods

### Study design, participants and methods

**Figure 1:**
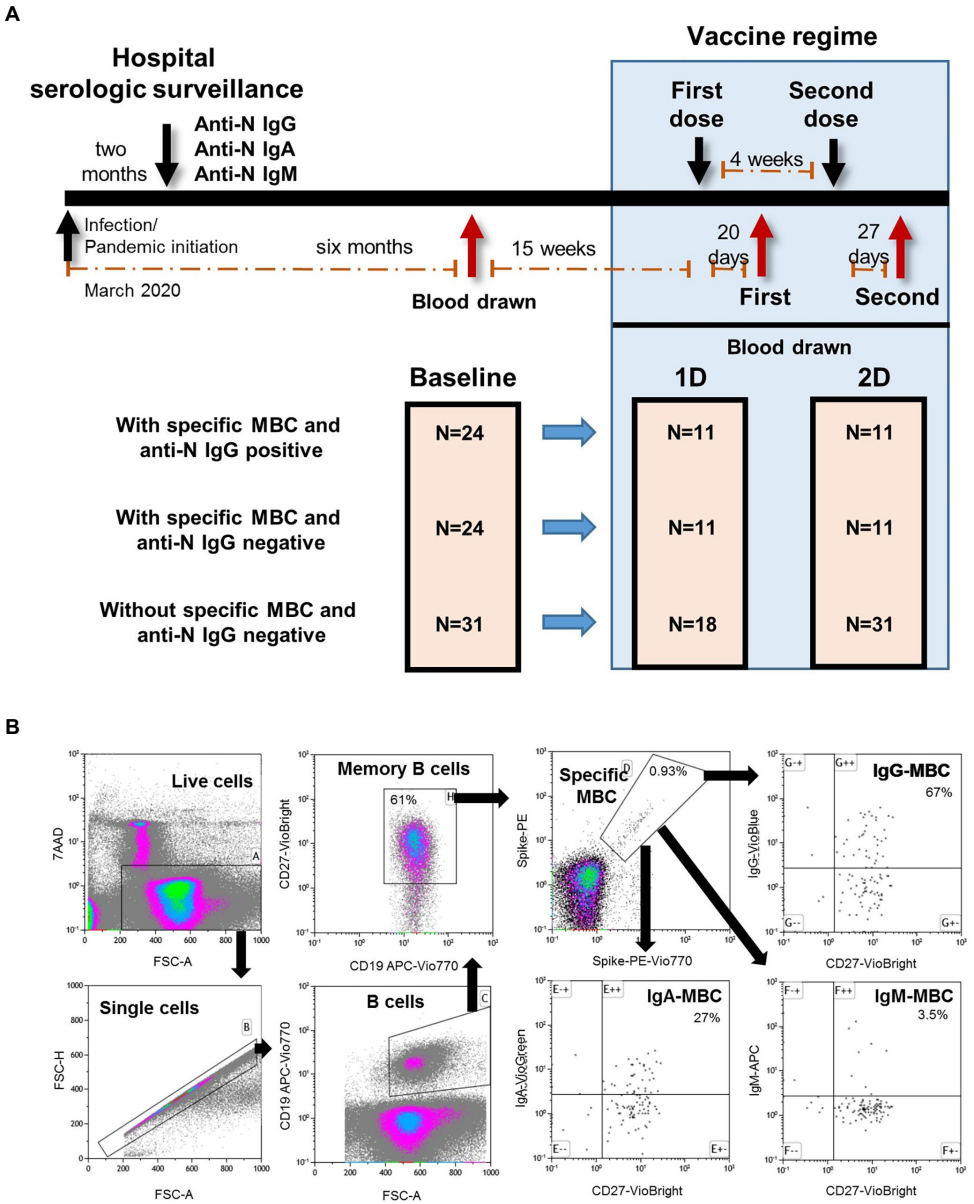
**(A)** Diagram of the longitudinal study of the well followed health care workers who received complete regimen of mRNA-based BNT162b2 SARS-CoV-2 vaccine. MBC, specific memory B cells; 1D, first dose; 2D, second dose. **(B)** Flow cytometry strategy for the quantification of SARS-CoV-2-specific memory B cells (MBC) of one representative individual of the IgG^+^MBC^+^ group. Viable cells (FSC-A/7AAA plot) were plotted with FSC-H/FSC-A parameters to exclude doublets. Single cells were then gated using CD19-APC-Vio770 and CD27-Vio-Bright-FITC to identify memory B cells. Spike-specific MBCs are then identify with a double staining with the two spike-tetramer on the diagonal of the dot plot. Finally, the use of IgG-VioBlue, IgA-VioGreen, and IgM-APC were used to quantify each specific isotype of spike-specific MBCs. Results are recorded as percentage among total memory B cells and isotypes among specific MBCs.

This longitudinal study included 79 Caucasian health care workers followed since March 2020 in the tertiary Ramon y Cajal University Hospital (Madrid, Spain) who were vaccinated with two doses of Comirnaty (mRNA-based BNT162b2, Pfizer-BioNTech) COVID-19 vaccine in February 2021. Twenty-four individuals had specific antibodies and 55 individuals were seronegative. The complete study design is shown in Figure 1A.

Peripheral blood mononuclear cells (PBMC) were isolated from EDTA-blood sample by Ficoll-Paque density gradient centrifugation using lymphocyte separation medium (Corning, New York, NY) and cryopreserved. Plasma samples were stored at −80°C.

All individuals included in the study provided either oral or written informed consent. This study was conducted in accordance with the Declaration of Helsinki (1996), approved by the institutional review boards of our Hospital Ethics Committee (EC162/20), and registered at the clinical trials repository.^1^ Demographic data including age, sex, race, body mass index (BMI), and comorbidities were recorded for all participants.

### Quantitative determination of anti-S IgG, IgA, and IgM antibodies

Participants were tested for anti-N SARS-CoV-2 IgG antibodies (COVID-19-SARS-CoV-2 IgG ELISA, Demeditech, Germany) at baseline and after median of 17 days after the first dose to confirm serologic status regardless of the antibody production upon the vaccine. Results were recorded as relative units per milliliter (U/mL), with a threshold of 11 U/ml.

They were also tested for anti-Spike IgG antibodies (SARS-CoV-2 IgG II Quant, Abbott, Maidenhead, United Kingdom) with a threshold of 50 arbitrary units per milliliter (AU/mL), and both anti-S IgA and anti-S IgM (COVID-19-SARS-CoV-2 IgA ELISA, COVID-19-SARS-CoV-2 IgM ELISA Demeditech, Germany), with a threshold of 0.1 U/ml at baseline and after both doses.

### Determination of antibodies that inhibit ACE2-RBD interaction

ACE2-RBD neutralizing antibodies were quantified using competitive inhibition enzyme immunoassay technique (Human Novel Coronavirus (SARS-CoV-2) Neutralizing Antibody ELISA Kit, MyBioSource) following the manufacturer’s instructions. Plate wells are pre-coated with SARS-CoV-2 RBD and horseradish peroxidase-conjugated ACE2 was added with the sample. The competitive inhibition reaction is launched between HRP-ACE2 and SARS-CoV-2 neutralizing antibodies in samples. A substrate solution is added to the wells and the color develops opposite to the amount of antibodies that inhibit ACE2-RBD interaction in the sample. Optical densities greater than half the optical density for the blank were considered negative. Results were recorded as ng/mL.

### Determination of SARS-CoV-2 spike-specific memory B cells

SARS-CoV-2-specific MBCs detection was performed by binding the recombinant spike protein to the respective antigen-specific B cell receptor (BCR) on circulating B cells (SARS-CoV-2 spike B cell analysis kit, Miltenyi Biotec, Germany) by multiparametric flow cytometry (Figure 1B for cytometry strategy). Tetramers formed from recombinant SARS-CoV-2 Spike-Prot (HEK)-Biotin with Streptavidin, PE and PE-Vio770, respectively, were used according to the manufacturer’s instructions. This quantitative and qualitative analysis of specific MBCs and isotypes was performed by single-cell flow cytometry with fluorochrome-conjugated antibodies, and the 7-AAD for the exclusion of dead and apoptotic cells using a minimum of 5×10^6^ PBMCs for each analysis. Results are recorded as percentage among total memory B cells and isotypes among specific MBCs.

### Determination of SARS-CoV-2 spike-specific T cells

**Figure 2:**
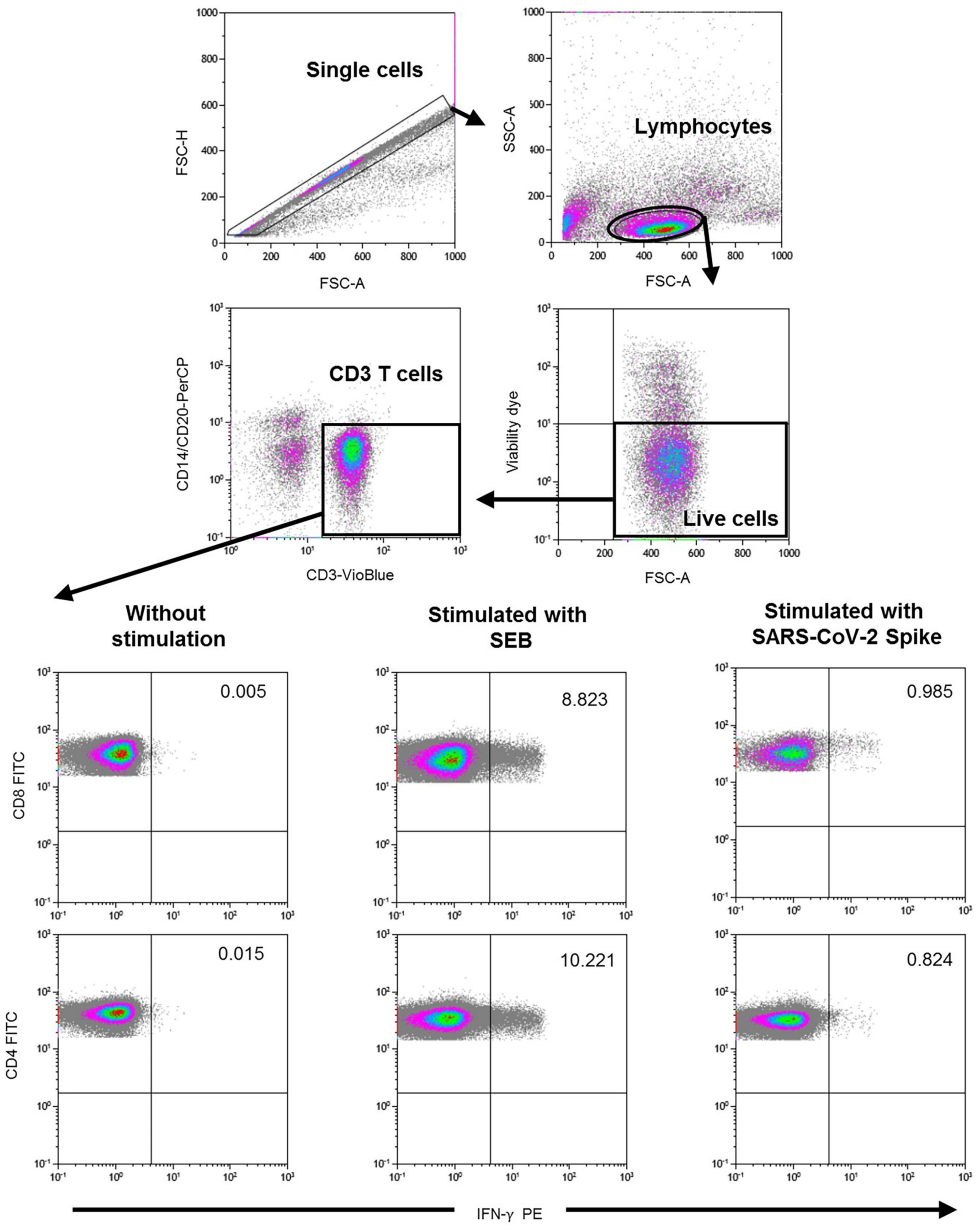
Flow cytometry gating strategy for the quantification of SARS-CoV-2 spike specific T cells. After stimulation with the spike peptide, staining of the cells was carried out with the following fluorochromes-conjugated antibodies: CD3-VioBlue, CD4-APC, CD8-FITC, CD14-PerCP, CD20-PerCP, IFN-γ-PE, and FcR blocking reagent. To exclude dead cells, viability 405/520 fixable dye staining was added for the last 10 min of incubation. Samples were measured and analyzed by flow cytometry on a MACSQuant Analyzer 10 using MACSQuantify software. At least 10^5^ cells were analyzed and gated with the following strategy: Single (FSC-A/FSC-H dot plot) and live cells were first selected. Cell debris, monocytes, and B cells were excluded from the analysis with CD14-and CD20-PerCP antibodies. Then, lymphocytes were selected with a FSC-A/SSC-A dot plot, and CD3 T cells were gated. IFN-γ expression was finally analyzed separately for CD4^+^ and CD8^+^ T cells. A representative sample of negative control (without stimulation), positive control (stimulated with SEB), and with SARS-CoV-2 Spike peptide are shown in the lower figure.

Specific CD4^+^ and CD8^+^ T cell responses were performed by intracellular cytokine staining using multiparametric flow cytometry. Briefly, after centrifugation at 200× *g* for 10 min, plasma fraction was collected and again centrifuged at 1200× *g* for 15 min, aliquoted and stored at −80°C. The cellular fraction was diluted with phosphate-buffered saline (PBS) and subjected to Ficoll density gradient centrifugation at 500× *g* for 20 min. PBMCs were washed and frozen in fetal bovine serum (FBS) with 8% dimethyl sulfoxide (DMSO, Sigma, United States) in liquid nitrogen. PBMCs were thawed and plated in 96-well flat-bottom plates at 10^6^ cells/well in RPMI-1640 culture medium (Gibco, United States) supplemented with 10% human serum (AB serum, Sigma), 100 IU of penicillin/streptomycin/mL (Gibco, United States), 2 mm L-glutamine, and after 24 h cells were stimulated in five different conditions in the presence of 1 μg/ml purified anti-CD28 antibody (Miltenyi, Germany). Three wells were stimulated with each of the SARS-CoV-2 peptide pools at a concentration of 1 μg/ml. Each peptide pool was composed of 15-mer sequences with 11 amino acids overlap, covering the immunodominant sequence domains of the surface glycoprotein spike (S) of SARS-CoV-2 (PepTivator SARS-CoV-2 Prot S, Miltenyi-Biotec, Cologne, Germany). In addition, one well was stimulated with culture medium alone as a negative control (unstimulated), and other well was stimulated adding 1.5 mg SEB (staphylococcal enterotoxin B, Sigma, Germany) as the positive control. An unresponsive sample to SEB would be excluded from the analysis. Stimulated PBMCs were incubated for 2 h before adding brefeldin A (Rapid Cytokine Inspector CD4/CD8 T cell kit, Miltenyi, Germany) into the medium to stop cytokine release and kept in culture for other 14 h. After stimulation, staining of the cells was carried out with the following fluorochromes-conjugated antibodies using Rapid Cytokine Inspector CD4/CD8 T cell kit (Miltenyi, Germany): CD3-VioBlue, CD4-APC, CD8-FITC, CD14-PerCP, CD20-PerCP, IFN-γ-PE, and FcR blocking reagent. To exclude dead cells, viability 405/520 fixable dye staining (Milteny, Germany) was added for the last 10 min of incubation. Fixation and permeabilization were performed according to the manufacturer’s protocol. Samples were measured and analyzed by flow cytometry on a MACSQuant Analyzer 10 using MACSQuantify software. At least 10^5^ cells were analyzed and gated with the following strategy. Single (FSC-A/FSC-H dot plot) and live cells were first selected. Cell debris, monocytes, and B cells were excluded from the analysis with CD14-and CD20-PerCP antibodies. Then, lymphocytes were selected with a FSC-A/SSC-A dot plot, and CD3 T cells were gated. IFN-γ expression was finally analyzed separately for CD4^+^ and CD8^+^ T cells and it was considered significant if there was at least 2-fold increase in reactive cells in comparison with the unstimulated control. The complete flow cytometry strategy is shown in Figure 2.

### Statistical analysis

Continuous variables were expressed as the median and interquartile range (IQ_25-75_) and categorical variables by frequencies and proportions. The Mann–Whitney *U* test (non-parametric) for independent samples was used to compare continuous variables. The Wilcoxon signed-rank test was used to compare paired samples to analyze the evolution of the measurements after vaccination. Spearman’s rank correlation coefficient was used to measure the association between two variables. Differences between categorical variables were evaluated using contingency tables (Chi-square distribution).

## Results

Individuals were included in three groups according with the detection of specific MBCs at baseline as follows: *IgG^−^MBC^−^group*, including 31 seronegative individuals without specific MBCs. They had not reported symptoms or positive serology since the beginning of the pandemic; *IgG^+^MBC^+^ group*, including 24 seropositive individuals with specific MBCs; and *IgG^−^MBC^+^ group*, including 24 seronegative individuals with specific MBCs. Among this last group, 9 reported nasopharyngeal swab RT-PCR positive test and/or anti-N IgG antibodies, 12 reported nasopharyngeal swab RT-PCR positive test without any record of seropositivity, one showed symptoms but never developed positive serology, and two never reported symptoms or positive serology. The baseline characteristics of the 79 health care workers included in the study are shown in Table 1. The IgG^−^MBC^−^group was older compared to the IgG^+^MBC^+^ group (*p* = 0.013) and the IgG^−^MBC^+^ group (*p* = 0.005). All three groups were similar in sex, body mass index, and time from COVID-19 diagnosis to inclusion in this study.

**Table 1:** Characteristics of the individuals included in this study.

	Anti-N IgG positive with specific MBC (IgG^+^MBC^+^)	Anti-N IgG negative with specific MBC (IgG^−^MBC^+^)	Anti-N IgG negative without specific MBC (IgG^−^MBC^−^)	ANOVA
	*N* = 24	*N* = 24	*N* = 31	*p*
Age (years)	42 [30–51]	39 [29–53]	51 [44–58]	0.006
Sex (Female)	14 (58.3%)	15 (62.5%)	22 (71%)	0.614
Body Mass Index				
Underweight (≤18.5)	0	0	1 (3.2%)	0.090
Normal weight (18.5–24.9)	22 (91.7%)	16 (66.7%)	20 (64.5%)	
Overweight (25–29.9)	2 (8.3%)	2 (8.3%)	8 (25.9%)	
Obesity (>30)	0	6 (25%)	2 (6.4%)	
Time from COVID-19 diagnosis to study inclusion (weeks)	26 [7–29]	26 [24–29]^*^	–	0.763
Serology at survey in May 2020				
Anti-N IgA/IgM positive	12	2	0	
Anti-N IgG positive	24	7	0	
Swab positive	16	17	0	
Serology at study in September–October 2020				
Anti-N IgG positive	24 (100%)	0	0	
Anti-S IgG positive	24 (100%)	0	0	
Anti-S IgA positive	8 (33.3%)	0	0	
Anti-S IgM positive	3 (12.5%)	0	0	

Data are expressed as median and interquartile range, and percentage. ANOVA for continuous and chi-square for categorical variables for statistical differences between variables. HCW, health care workers; IgA antibodies by ELISA and IgG and IgM antibodies by rapid chromatographic immunoassay. MBC, memory B cells.

* Two individuals were never diagnosed.

### Levels of specific memory B cells (MBC) and association with antibodies at baseline

**Figure 3:**
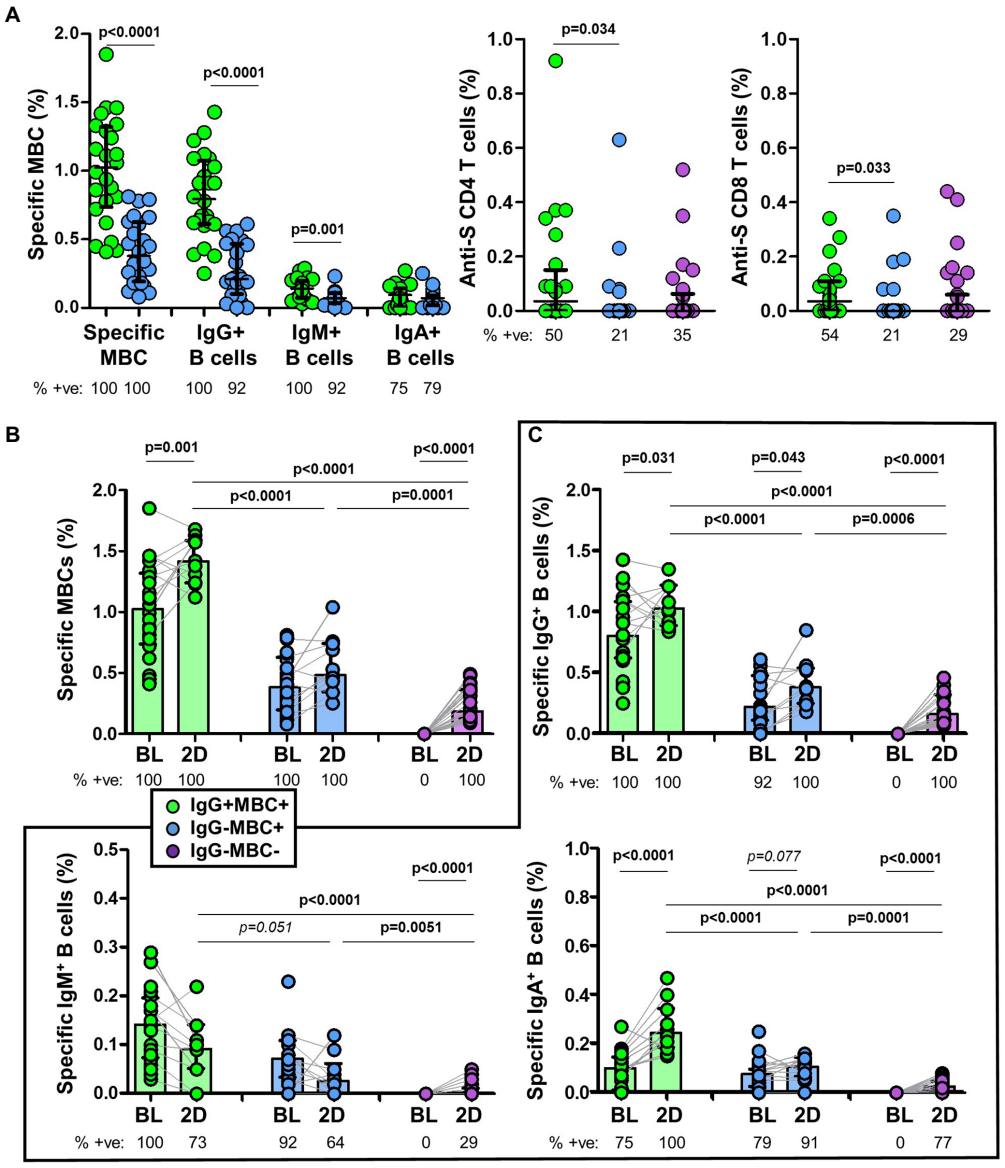
**(A)** Frequency of specific memory B cells (MBCs) and isotypes in IgG^+^MBC^+^ (green) and IgG^−^MBC^+^ (blue) groups, and anti-S CD4^+^ and CD8^+^ T cells in IgG^+^MBC^+^ (green), IgG^−^MBC^+^ (blue), and IgG^−^MBC^−^ (purple) groups after two doses of vaccine. **(B)** Frequency of specific MBCs after two doses of vaccine compared to baseline in IgG^+^MBC^+^ (green), IgG^−^MBC^+^ (blue), and IgG^−^MBC^−^ (purple) groups. **(C)** Frequency of isotype IgG, IgA and IgM memory B cells after two doses of vaccine compared to baseline in IgG^+^MBC^+^ (green), IgG^−^MBC^+^ (blue), and IgG^−^MBC^−^ (purple) groups. Mann–Whitney test. Only significant differences are shown. Statistically significant when *p* < 0.05.

**Figure 4:**
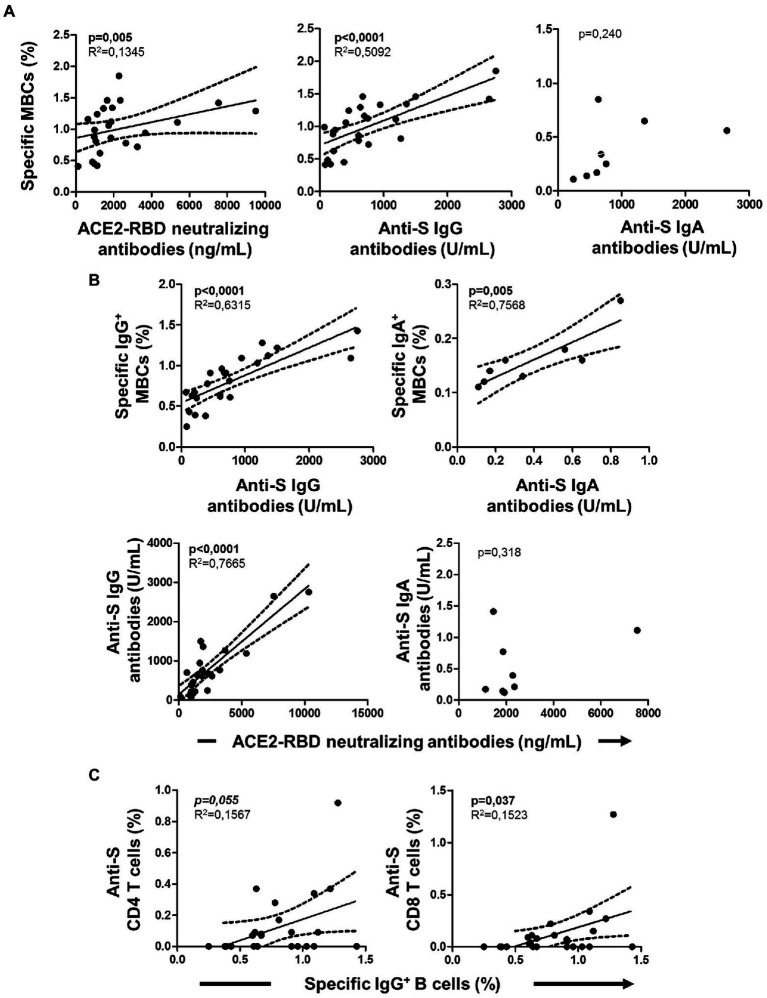
Associations of cellular immunity with specific serology at baseline in IgG^+^MBC^+^ group. **(A)** Association of specific memory B cells (MBCs) with ACE2-RBD neutralizing antibodies, anti-S IgG and anti-S IgA antibodies. **(B)** Association of specific isotype IgG and IgA MBCs with anti-S IgG and anti-S IgA antibodies, respectively. Association of ACE2-RBD neutralizing antibodies with anti-S IgG and anti-S IgA antibodies. **(C)** Association of IgG^+^ MBCs with anti-S CD4 and CD8 T cells. Pearson’s correlation *r* value and two-tailed *p* values are shown only when statistically significant (*p* < 0.05).

Among individuals with specific MBCs, those with positive serology (IgG^+^MBC^+^ group) had higher levels of specific MBCs (*p* < 0.0001), IgG^+^ B cells (*p* < 0.0001), and IgM^+^ B cells (*p* = 0.001) compared to individuals without positive serology (IgG^−^MBC^+^ group). The level of IgA^+^ B cells was similar among the above two groups (*p* = 0.135), as shown in Figure 3A. Specific MBCs positively correlated with ACE2-RBD neutralizing antibodies (*p* = 0.077, although not significant) and anti-S IgG antibodies (*p* < 0.0001) among IgG^+^MBC^+^ individuals at baseline, as shown in Figure 4A. Specific MBCs were not associated with the level of anti-S IgA antibodies (*p* = 0.241), and the correlation with the level of anti-S IgM could not be performed due to the limited number of antibody positive individuals (N = 3). In parallel, specific IgG^+^ B cells correlated with the level of anti-S IgG antibodies (*p* < 0.0001) and specific IgA^+^ B cells correlated with the level of anti-S IgA antibodies (*p* = 0.005), as shown in Figure 4B. On the other hand, only, anti-S IgG antibodies positively correlated with the level of ACE2-RBD neutralizing antibodies (*p* < 0.0001). Specific memory B cells positively correlated with anti-S CD4 (*p* = 0.055) and CD8 (*p* = 0.037) T cells, as shown in Figure 4C.

### Association between specific MBC and antibodies after vaccination

Specific MBCs increased in all individuals after the second dose of the vaccine (Figure 3B). Interestingly, the level of specific MBCs in IgG^−^MBC^−^group was lower compared to that found in IgG^−^MBC^+^ (*p* = 0.0001) and IgG^+^MBC^+^ (*p* < 0.0001) groups, respectively.

Since specific IgG^+^ B cells were developed in all individuals (Figure 3C), the level of these cells was significantly lower in IgG^−^MBC^−^group compared to the other two groups. Only 77% of the individual in IgG^−^MBC^−^group developed specific IgA^+^ B cells and again with lower levels. The expansion of specific IgM^+^ B cells was very limited especially in IgG^−^MBC^+^ and IgG^−^MBC^−^groups, reaching only 29% in the last group.

**Figure 5:**
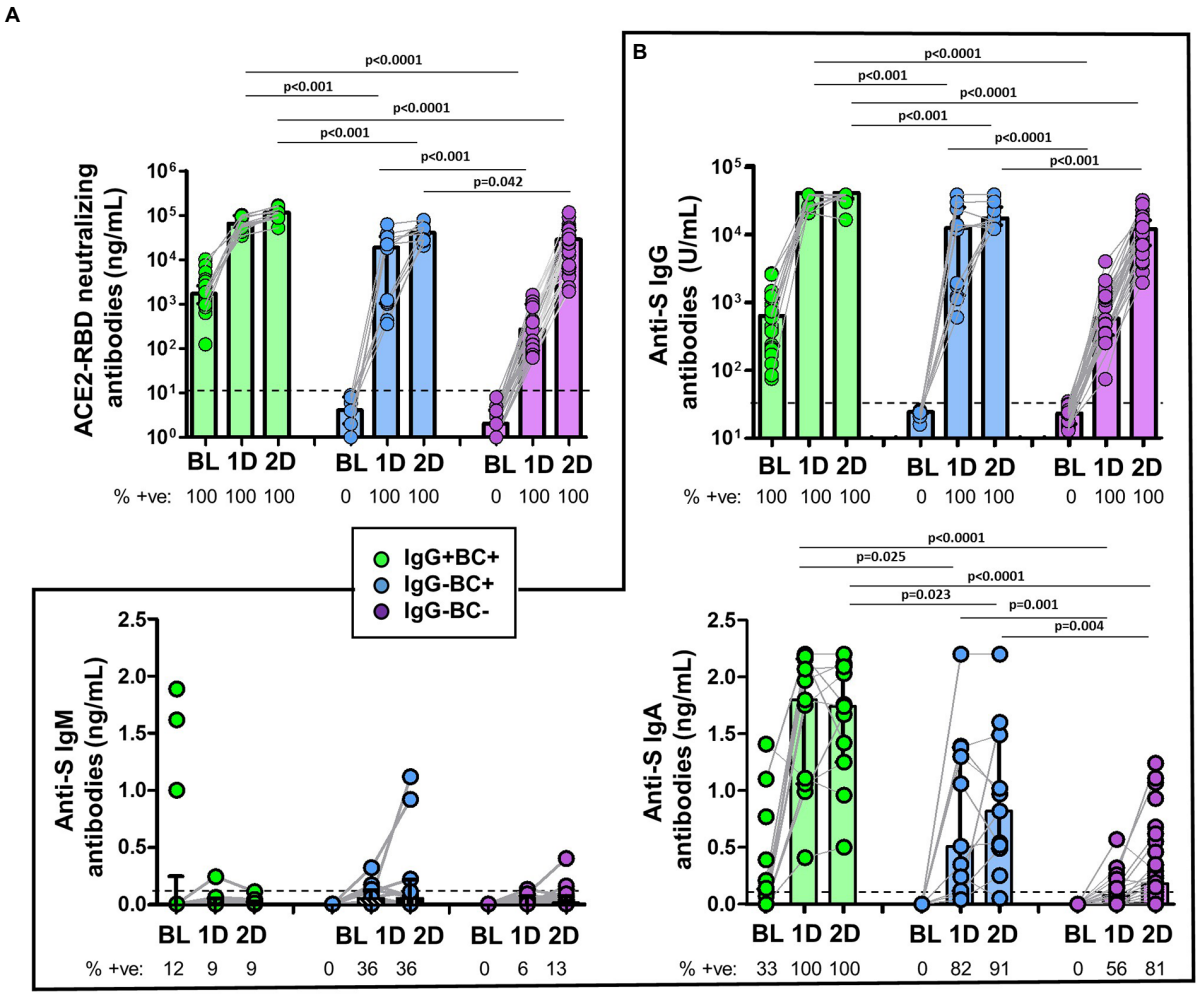
**(A)** Comparison of the levels of ACE2-RBD neutralizing antibodies in IgG^+^MBC^+^ (green), IgG^−^MBC^+^ (blue), and IgG^−^MBC^−^ (purple) groups at baseline and after the first and second doses of the vaccine. The percentage of positive individuals are shown under the figure. Dotted line represents the positivity cut-off. **(B)** Comparison of the levels of anti-S IgG, IgM and IgA antibodies in IgG^+^MBC^+^ (green), IgG^−^MBC^+^ (blue), and IgG^−^MBC^−^ (purple) groups at baseline and after the first and second doses of the vaccine. The percentage of positive individuals are shown under the figure. Dotted line represents the positivity cut-off. Mann–Whitney test. Only statistically significant values (*p* < 0.05) are shown.

ACE2-RBD neutralizing antibodies and anti-S IgG antibodies were detected in all individuals (Figures 5A,B). Interestingly, ACE2-RBD neutralizing antibodies in IgG^−^MBC^−^group were significantly lower compared to that found in both IgG^−^MBC^+^ and IgG^+^MBC^+^ groups after the first and the second dose (Figure 5A). As expected, IgG^−^MBC^+^ group showed lower levels of ACE2-RBD neutralizing antibodies compared to IgG^+^MBC^+^ group after the first (*p* < 0.001) and the second dose (*p* < 0.0001). The level of anti-S IgG in IgG^−^MBC^−^group was lower compared to the other two groups (Figure 5B). After the first dose, anti-S IgA antibodies were detected in 56% (10/18) of the individuals in IgG^−^MBC^−^group, while in 82 and 100% in IgG^−^MBC^+^ and IgG^+^MBC^+^ groups, respectively. The level of anti-S IgA antibodies was lower in IgG^−^MBC^−^group compared to the other groups. After the second dose, 81% of the individuals in IgG^−^MBC^−^group had anti-S IgA antibodies, while 91 and 100% were detected in IgG^−^MBC^+^ and IgG^−^MBC^−^groups, respectively. Again, the level of antibodies was lower in the IgG^−^MBC^−^group compared to the other groups. Anti-S IgM antibodies were barely detected after the first and the second doses among the three groups and the level was very low in all of them.

### Association between specific MBC and T cells

**Figure 6:**
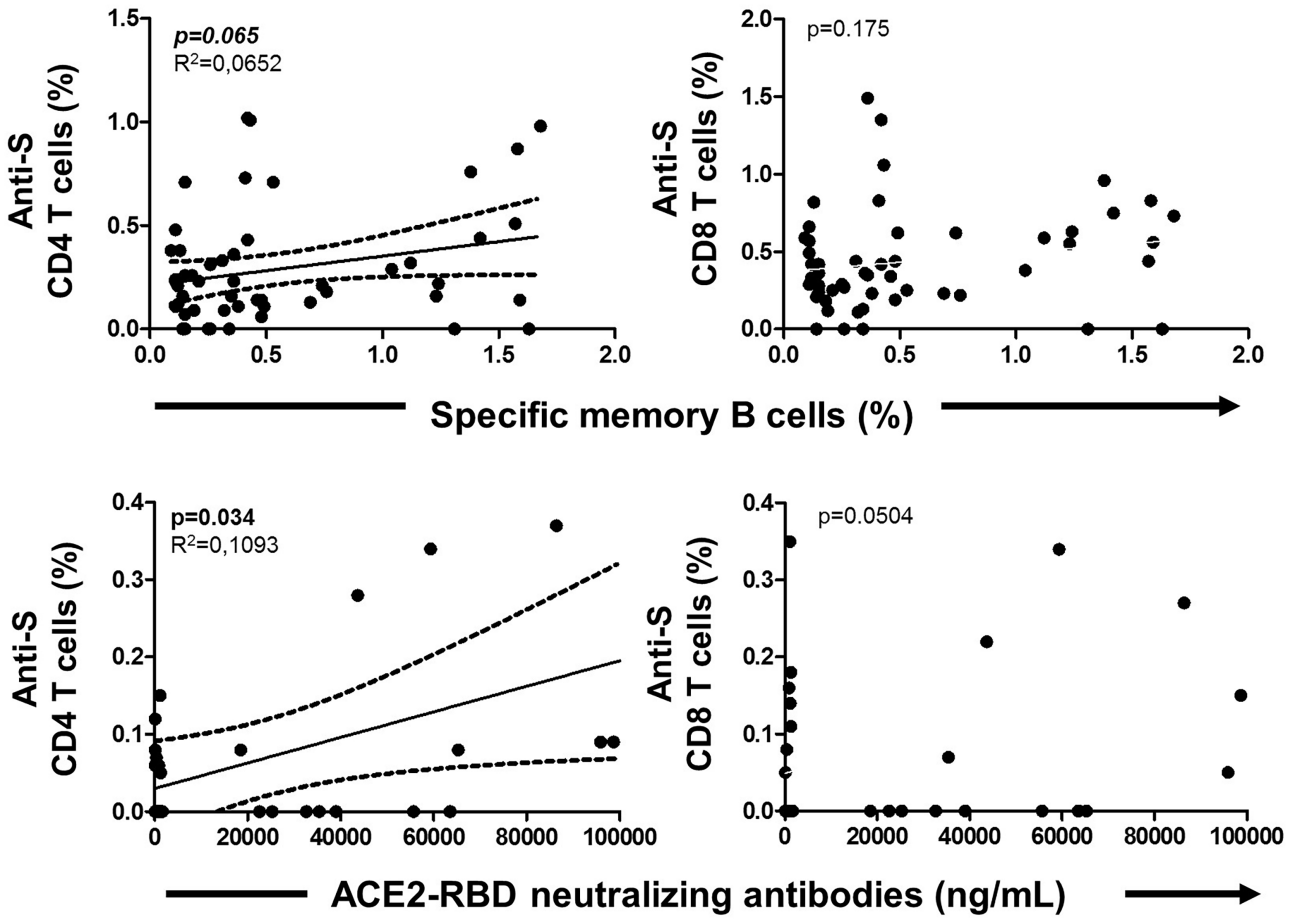
Association of specific memory B cells with anti-S CD4 and CD8 T cells (upper side) and ACE2-RBD neutralizing antibodies with anti-S CD4 and CD8 T cells (lower side) after vaccination. Pearson’s correlation *r* value and two-tailed *p* values are shown only with statistical significance (*p* < 0.05).

**Figure 7:**
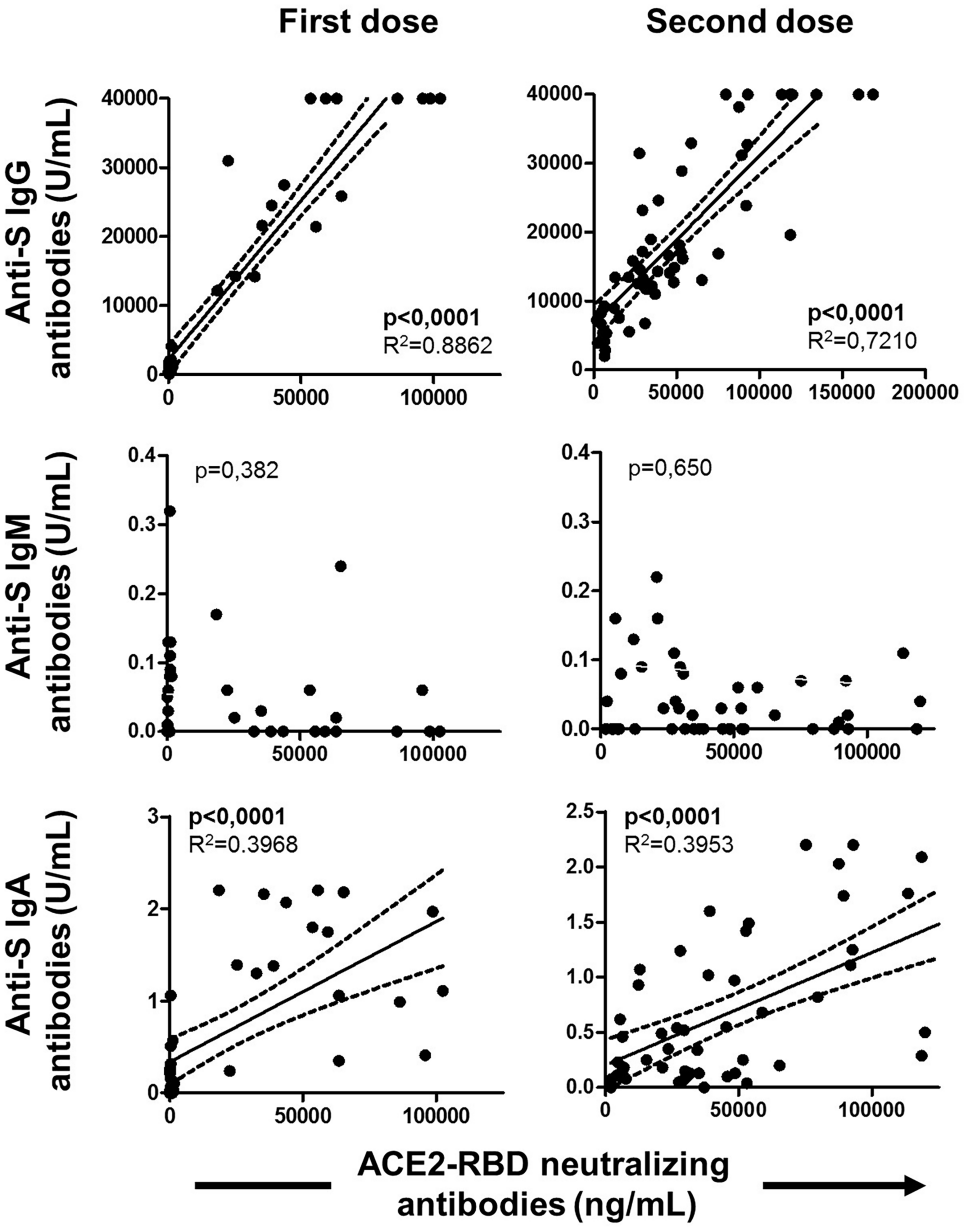
Associations of ACE2-RBD neutralizing antibodies with anti-S IgG, IgM and IgA antibodies after the first and the second doses of the vaccine. Pearson’s correlation *r* value and two-tailed *p* values are shown only with statistical significance (*p* < 0.05).

**Figure 8:**
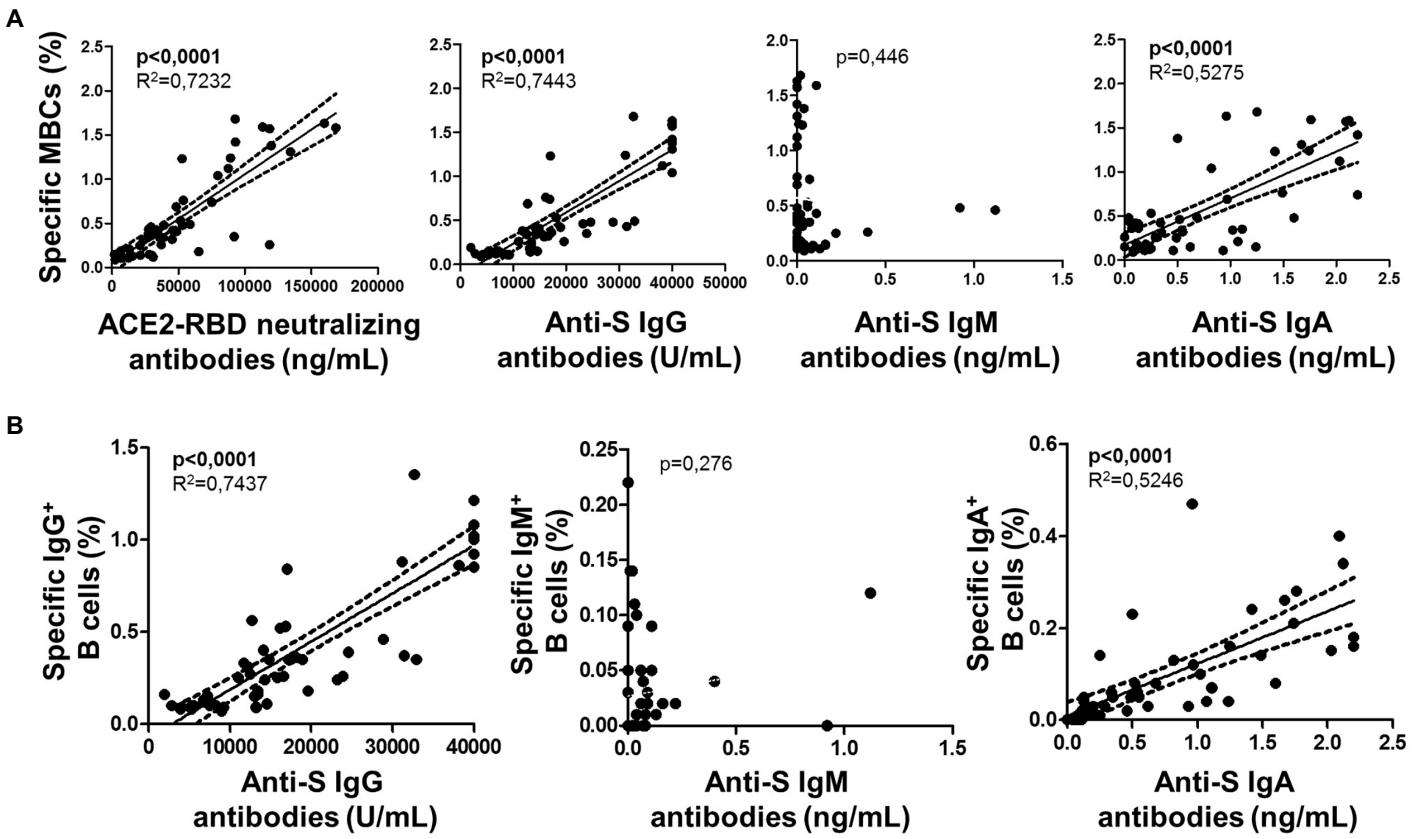
**(A)** Association of specific memory B cells (MBCs) with ACE2-RBD neutralizing antibodies, anti-S IgG, IgM and IgA antibodies after the complete vaccine regimen. **(B)** Associations of specific IgG B cells with anti-S IgG, IgM and IgA antibodies. Pearson’s correlation *r* value and two-tailed *p* values are shown only with statistical significance (*p* < 0.05).

The specific T cell response was partially published elsewhere by our group and here we found it associated with the level of specific MBCs. Hence, specific MBCs directly correlated with specific CD4^+^ T cells (although not significant, *p* = 0.065), while no correlation was found with specific CD8^+^ T cells (*p* = 0.156) after vaccination, as shown in Figure 6. In parallel, ACE2-RBD neutralizing antibodies positively correlated with specific CD4^+^ T cells (*p* = 0.034), but not with CD8^+^ T cells (*p* = 0.158). ACE2-RBD neutralizing antibodies positively correlated with anti-S IgG and IgA antibodies after the first (*p* < 0.0001) and second (*p* < 0.0001), as shown in Figure 7. In the same way, specific MBC (*N* = 53) positively correlated with ACE2-RBD neutralizing antibodies (*p* < 0.0001), anti-S IgG (*p* < 0.0001), and anti-S IgA (*p* < 0.0001) antibodies after the second dose (Figure 8A). After the second dose, IgG^+^ B cells positively correlated with anti-S IgG antibodies (*p* < 0.0001), while IgA^+^ B cells positively correlated with anti-S IgA antibodies (*p* < 0.0001), as shown in Figure 8B.

### Association between specific unswitched and switched MBCs

**Figure 9:**
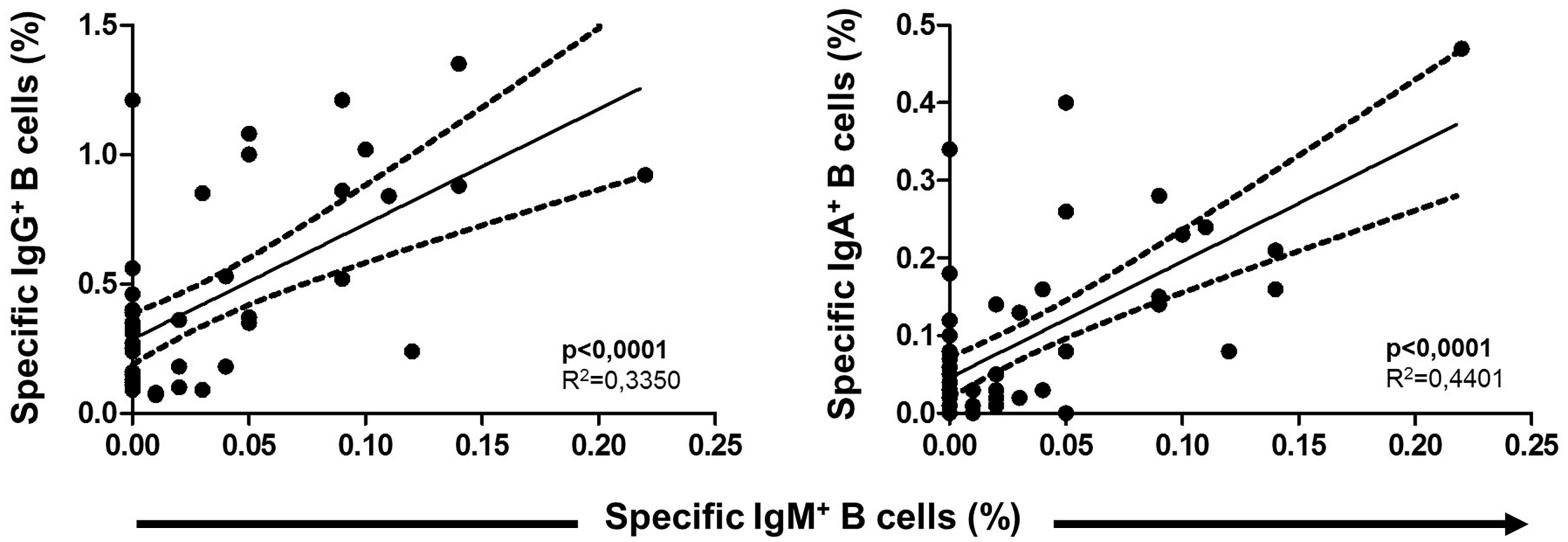
Association between specific IgM^+^ B cells (unswitched) with both switched IgG^+^ and IgA^+^ B cells. Pearson’s correlation *r* value and two-tailed *p* values are shown.

Unswitched IgM^+^ MBCs strongly correlated positively with both switched isotypes IgG^+^ and IgA^+^ MBCs (*p* < 0.001 in both cases) after vaccination, as shown in Figure 9. On the other hand, no correlation between overall MBCs with both specific MBCs and isotypes of MBC were found.

### Vaccine breakthrough

**Figure 10:**
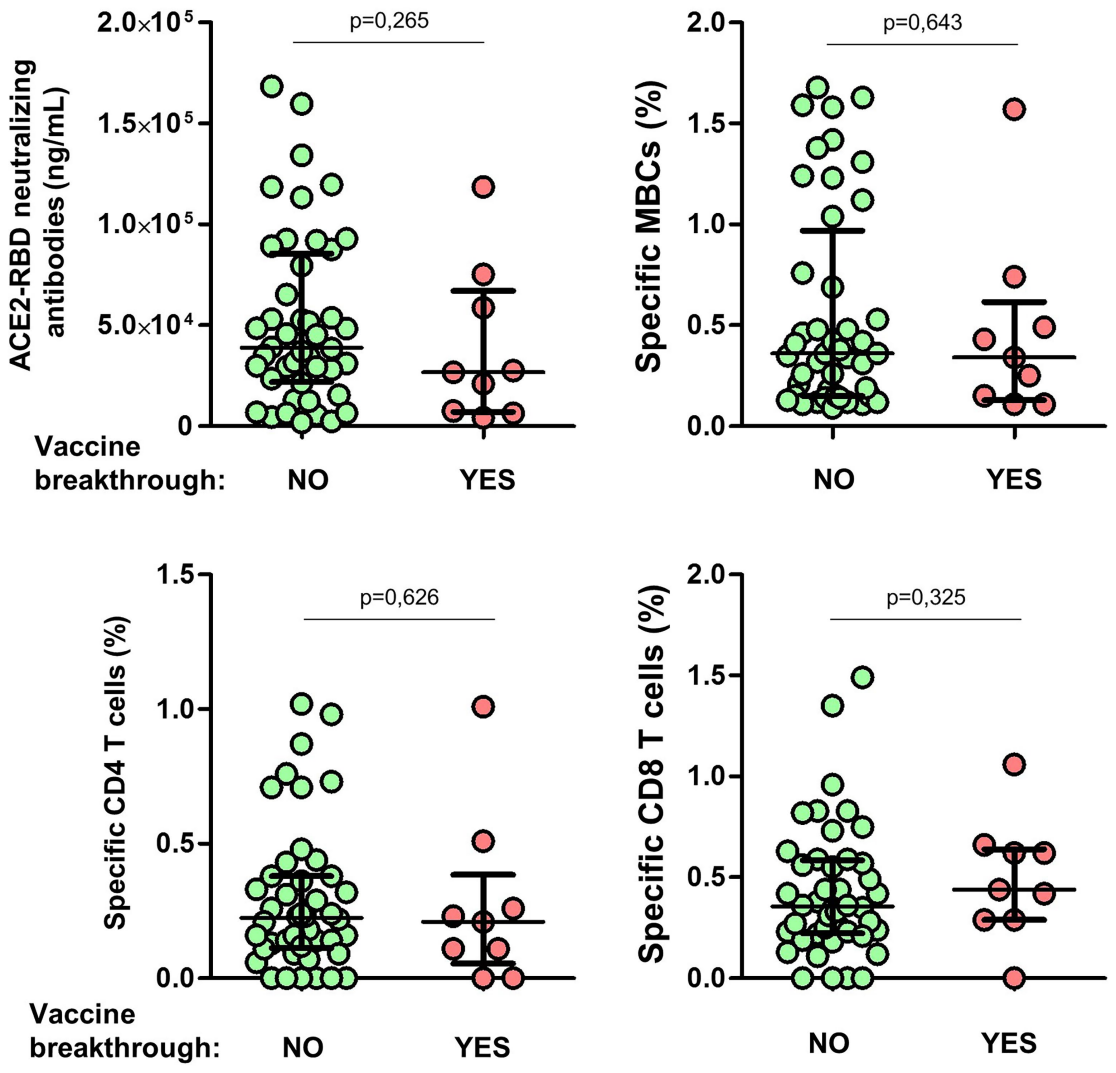
Differences of ACE2-RBD neutralizing antibodies, specific memory B (MBCs) and T cells levels according to subsequent vaccine breakthrough. Mann–Whitney test.

All individuals were followed for nearly 1 year after the complete vaccine regimen. Eight individuals (17%) became infected during the Omicron wave (between late 2021 and early 2022), all with mild symptoms. The level of ACE2-RBD neutralizing antibodies after the second dose was similar in individuals with or without vaccine breakthroughs, as shown in Figure 10. In addition, no differences in specific MBC, CD4^+^ or CD8^+^ T cells were found between individuals with or without vaccine breakthroughs.

## Discussion

One of the current concerns about immune protection after SARS-CoV-2 infection or vaccination is the fact that specific antibodies wane between a few months (Ibarrondo et al., 2020; Long et al., 2020; Casado et al., 2021) and up to 6 months after infection (Lozano-Rodríguez et al., 2020; Wajnberg et al., 2020; Cui et al., 2021; Gaebler et al., 2021; Sherina et al., 2021; Zuo et al., 2021; Chen et al., 2022) due to the reduction of short-lived plasma cells. Although this has been seen as a sign of loss of immune protection, a pool of cells including memory B cells (MBC), long-lived plasma cells and memory T cells are key cells for keeping the protective immune memory on alert, despite the absence of circulating specific antibodies. The absence of these cells would make the immune response worse after an eventual infection.

We reported here that seronegative individuals without specific MBCs developed lower humoral and cellular (including specific T and B cell responses) after two doses of mRNA-based Comirnaty vaccine, compared to individuals with specific MBCs with or without specific antibodies. Of note, in the absence of specific antibodies, the presence of specific MBCs acts as booster for potentially rapid and high-quality protective immune response.

In accordance with other studies (Mortari et al., 2021; Balachandran et al., 2022), we found specific MBC in convalescents with undetectable specific antibodies before vaccination but with lower levels compared to seropositive individuals with specific MBCs before vaccination, especially isotypes IgG^+^ and IgM^+^ B cells. Nevertheless, this lower level was enough to generate faster and higher levels of ACE2-RBD neutralizing antibodies and anti-S switched isotypes (anti-S IgG and IgA antibodies) early after the first dose of the vaccine compared to seronegative individuals without specific MBCs.

After the complete vaccine regimen, although all IgG^−^MBC^−^individuals developed specific IgG^+^ MBCs, anti-S IgG, and ACE2-RBD neutralizing antibodies, only 77% had detectable IgA^+^ MBC, and 81% developed anti-S IgA antibodies. This was in parallel with lower levels of specific MBC or antibodies compared to the other two groups. In contrast with other groups where most of the individuals showed IgM^+^ B cell isotype after vaccination (Mortari et al., 2021), in our study, only 29% of the IgG^−^MBC^−^individuals showed this cellular isotype. This suggests again that the absence of specific MBCs and antibodies before vaccination, or after a period of time after vaccination when specific responses waned and lost, correlates with lower protection against an eventual infection. Therefore, these individuals may benefit from a vaccine booster. It is also interesting that IgM+ B cell isotype positively correlated with switched (IgA^+^ and IgG^+^) B cell isotypes. A stronger initial B cell response (unswitched isotype) drive to the development of high-affinity switched isotypes, conferring a stronger B cell response. On the contrary, the level of overall memory B cells did not correlate with the level of specific memory B cells, including both unswitched and switched isotypes.

Very few published studies have compared specific antibodies, MBCs, CD8^+^ and CD4^+^ T cells following vaccination in the same individuals. Whether these specific T cells could serve as surrogates for protective immunity remains to be determined. Data on specific CD4^+^ and CD8^+^ T cells of the individuals included in this study have been reported previously (Casado et al., 2021, 2022; Cortés et al., 2022). In agreement with others (Mortari et al., 2021; Zollner et al., 2021), we identified specific MBCs associated with specific CD4^+^ T cells (although not significant), while we did not find an association with specific CD8^+^ T cells found by others (Chen et al., 2022; Vitiello et al., 2022), indicating a partial convergent development of humoral and cellular adaptive immunity in our study. In contrast with other studies (Chen et al., 2022), specific CD4^+^ T cells did not correlate with specific antibodies perhaps because we analyzed total specific CD4^+^ T cells rather than subsets such as T follicular helper (Tfh) cells. Some studies have suggested that CD4^+^ Tfh responses are key in SARS-CoV-2 infection (Cui et al., 2021). However, the characteristics of these Tfh responses at, or soon after, infection have not been related to the specific MBCs and antibody responses for SARS-CoV-2 1 year after infection.

Maintenance of high-quality protective response is becoming increasingly important with the ongoing development and transmission of new variants of concern (VoCs). Although the precise titer of neutralizing antibodies required to infer protection is unclear, even low titers of neutralizing antibodies in humans have been associated with protection against severe disease (Purtha et al., 2011; Chen et al., 2022; Kaplonek et al., 2022). Eight individuals (17%) became infected after 1 year of the complete vaccine regimen during the Omicron wave (between late 2021 and early 2022), although with mild symptoms. Unfortunately, the titer of ACE2-RBD neutralizing antibodies and the level of specific MBCs after the second dose of the vaccine were similar in individuals with or without vaccine breakthrough, not supporting the association of these immune responses with protection against infection at least 1 year earlier. Nevertheless, this has to be taken with caution since the number of individuals infected after vaccination was very low and hence the power of this analysis is limited.

One limitation of this study was the limited number of individuals included who completed the two doses of the vaccine regimen. Nevertheless, all these individuals have been followed very closely since the beginning of the pandemic are have been very well characterized.

In conclusion, seronegative individuals with undetectable specific MBCs showed the worst humoral and cellular responses, both in frequency and magnitude. These individuals are less protected and hence are good candidates for earlier boosting vaccines to prevent eventual infections or at least to prevent severe disease compared to seronegative individuals with specific memory B cells. Monitoring specific T cell response is nowadays implemented as a clinical resource in certain hospitals in Spain. According to our results, monitoring specific B cells should be also implemented in hospital given its potential clinical relevance and the great help for clinicians to identify those individuals that may require more close evaluations and vaccine boosters. This assay could be implemented without much technical difficulty since it only requires a flow cytometry analysis, without the need for cell cultures.

## Data availability statement

The raw data supporting the conclusions of this article will be made available by the authors, without undue reservation.

## Ethics statement

The studies involving human participants were reviewed and approved by Ramón y Cajal Ethics Committee (EC162/20). The patients/participants provided their written informed consent to participate in this study.

## Author contributions

JC and AV designed the conceptual framework of the study and analyzed the data. AM-H, HV, and AV performed all the flow cytometry experiments. AM-H and HV performed the sample processing. JC, JH, and PV helped in the recruitment of all the individuals. SG-M helped in the collection of blood samples. All authors contributed to the article and approved the submitted version.

## Funding

This study was internally funded by the departmental funds available to JC and the authors received no specific funding for this work.

## Conflict of interest

The authors declare that the research was conducted in the absence of any commercial or financial relationships that could be construed as a potential conflict of interest.

## Publisher’s note

All claims expressed in this article are solely those of the authors and do not necessarily represent those of their affiliated organizations, or those of the publisher, the editors and the reviewers. Any product that may be evaluated in this article, or claim that may be made by its manufacturer, is not guaranteed or endorsed by the publisher.

## Footnotes

^1^clinicaltrials.gov, NCT04402827

## References

[ref1] Abayasingam A. Balachandran H. Agapiou D. Hammoud M. Rodrigo C. Keoshkerian E.. (2021). Long term persistence of RBD-positive memory B cells encoding neutralizing antibodies in SARS-CoV-2 infection. Cell Rep. Med. 2:100228. doi: 10.1016/j.xcrm.2021.100228, PMID: 33748788 PMC7955929

[ref2] Balachandran H. Phetsouphanh C. Agapiou D. Adhikari A. Rodrigo C. Hammoud M.. (2022). Maintenance of broad neutralizing antibodies and memory B cells 1 year post-infection is predicted by SARS-CoV-2-specific CD4+ T cell responses. Cell Rep. 38:110345. doi: 10.1016/j.celrep.2022.110345, PMID: 35090598 PMC8768427

[ref3] Casado J. L. Haemmerle J. Vizcarra P. Rodríguez-Domínguez M. Velasco T. Velasco H.. (2021). T-cell response after first dose of BNT162b2 SARS-CoV-2 vaccine amongst health care workers with previous infection or cross-reactive immunity. Clin. Transl. Immunol. 10:e1341. doi: 10.1002/cti2.1341, PMID: 34522381 PMC8426108

[ref4] Casado J. L. Vizcarra P. Haemmerle J. Velasco H. Martín-Hondarza A. Rodríguez-Domínguez M. J.. (2022). Pre-existing T cell immunity determines the frequency and magnitude of cellular immune response to two doses of mRNA vaccine against SARS-CoV-2. Vaccine X 11:100165. doi: 10.1016/j.jvacx.2022.100165, PMID: 35529539 PMC9057925

[ref5] Casado J. L. Vizcarra P. Velasco H. Häemmerle J. McGee A. Fernández-Escribano M.. (2021). Progressive and parallel decline of humoral and T cell immunity in convalescent health care workers with asymptomatic or mild-moderate SARS-CoV-2 infection. J. Infect. Dis. 224, 241–245. doi: 10.1093/infdis/jiab242, PMID: 33961690 PMC8136002

[ref6] Chen Y. Yin S. Tong X. Tao Y. Ni J. Pan J.. (2022). Dynamic SARS-CoV-2-specific B-cell and T-cell responses following immunization with an inactivated COVID-19 vaccine. Clin. Microb. Infect. 28, 410–418. doi: 10.1016/j.cmi.2021.10.006, PMID: 34715346 PMC8547974

[ref7] Cortés A. Casado J. L. Longo F. Serrano J. J. Saavedra C. Velasco H.. (2022). Limited T-cell response to mRNA SARS-CoV-2 mRNA vaccine among patients with cancer receiving different cancer treatments. Eur. J. Cancer 166, 229–239. doi: 10.1016/j.ejca.2022.02.017, PMID: 35316750 PMC8885286

[ref8] Cui D. Tang Y. Jiang Q. Jiang D. Zhang Y. Lv Y.. (2021). Follicular helper T cells in the immunopathogenesis of SARS-CoV-2 infection. Front. Immunol. 12:731100. doi: 10.3389/fimmu.2021.731100, PMID: 34603308 PMC8481693

[ref9] Dan J. M. Mateus J. Kato Y. Hastie K. M. Yu E. D. Faliti C. E.. (2021). Immunological memory to SARS-CoV-2 assessed for up to 8 months after infection. Science 371:eabf4063. doi: 10.1126/science.abf4063, PMID: 33408181 PMC7919858

[ref10] Gaebler C. Wang Z. Lorenzi J. C. C. Muecksch F. Finkin S. Tokuyama M.. (2021). Evolution of antibody immunity to SARS-CoV-2. Nature 591, 639–644. doi: 10.1038/s41586-021-03207-w, PMID: 33461210 PMC8221082

[ref11] Giannotta G. Giannotta N. (2021). mRNA COVID-19 vaccines and long-lived plasma cells: a complicated relationship. Vaccine 9:1503. doi: 10.3390/vaccines9121503, PMID: 34960249 PMC8703557

[ref12] Hartley G. E. Edwards E. S. J. Aui P. M. Varese N. Stojanovic S. McMahon J.. (2020). Rapid generation of durable B cell memory to SARS-CoV-2 spike and nucleocapsid proteins in COVID-19 and convalescence. Sci. Immunol. 5:eabf8891. doi: 10.1126/sciimmunol.abf8891, PMID: 33443036 PMC7877496

[ref13] Ibarrondo F. J. Fulcher J. A. Goodman-Meza D. Elliott J. Hofmann C. Hausner M. A.. (2020). Rapid decay of anti–SARS-CoV-2 antibodies in persons with mild COVID-19. N. Engl. J. Med. 383, 1085–1087. doi: 10.1056/NEJMc2025179, PMID: 32706954 PMC7397184

[ref14] Kaplonek P. Cizmeci D. Fischinger S. Collier A. R. Suscovich T. Linde C.. (2022). mRNA-1273 and BNT162b2 COVID-19 vaccines elicit antibodies with differences in Fc-mediated effector functions. Sci. Transl. Med. 14:eabm2311. doi: 10.1126/scitranslmed.abm2311, PMID: 35348368 PMC8995030

[ref15] Long Q. X. Tang X. J. Shi Q. L. Li Q. Deng H. J. Yuan J.. (2020). Clinical and immunological assessment of asymptomatic SARS-CoV-2 infections. Nat. Med. 26, 1200–1204. doi: 10.1038/s41591-020-0965-6, PMID: 32555424

[ref16] Lozano-Rodríguez R. Valentín-Quiroga J. Avendaño-Ortiz J. Martín-Quirós A. Pascual-Iglesias A. Terrón-Arcos V.. (2020). Cellular and humoral functional responses after BNT162b2 mRNA vaccination differ longitudinally between naive and subjects recovered from COVID-19. Cell Rep. 38:110235. doi: 10.1016/j.celrep.2021.110235, PMID: 34986327 PMC8687760

[ref17] Mortari E. P. Russo C. Vinci M. R. Terreri S. Fernandez Salinas A. Piccioni L.. (2021). Highly specific memory B cells generation after the 2nd dose of BNT162b2 vaccine compensate for the decline of serum antibodies and absence of mucosal IgA. Cells 10:2541. doi: 10.3390/cells10102541, PMID: 34685521 PMC8533837

[ref18] Ponticelli D. Antonazzo I. C. Caci G. Vitale A. Ragione G. D. Romano M. L.. (2021). Dynamics of antibody response to BNT162b2 mRNA COVID-19 vaccine after 6 months. J. Travel Med. 28:taab173. doi: 10.1093/jtm/taab173, PMID: 34697627

[ref19] Purtha W. E. Tedder T. F. Johnson S. Bhattacharya D. Diamond M. S. (2011). Memory B cells, but not long-lived plasma cells, possess antigen specificities for viral escape mutants. Exp. Med. 208, 2599–2606. doi: 10.1084/jem.20110740, PMID: 22162833 PMC3244041

[ref20] Reyes R. A. Clarke K. Gonzales S. J. Cantwell A. M. Garza R. Catano G.. (2021). SARS-CoV-2 spike-specific memory B cells express markers of durable immunity after non-severe COVID-19 but not after severe disease. bioRxiv preprint. doi: 10.1101/2021.09.24.461732, PMID: 34936684 PMC8694470

[ref21] Sherina N. Piralla A. Du L. Wan H. Kumagai-Braesch M. Andrell J.. (2021). Persistence of SARS-CoV-2–specific B and T cell responses in convalescent COVID-19 patients 6-8 months after the infection. Med. 2, 281–95.e4. doi: 10.1016/j.medj.2021.02.001, PMID: 33589885 PMC7874960

[ref22] Sokal A. Chappert P. Barba-Spaeth G. Roeser A. Fourati S. Azzaoui I.. (2021). Maturation and persistence of the anti-SARS-CoV-2 memory B cell response. Cells 184:e14, 1201–1213.e14. doi: 10.1016/j.cell.2021.01.050, PMID: 33571429 PMC7994111

[ref23] Tsatsakis A. Vakonaki E. Tzatzarakis M. Flamourakis M. Nikolouzakis T. K. Poulas K.. (2021). Immune response (IgG) following full inoculation with BNT162b2 COVID19 mRNA among healthcare professionals. Int. J. Mol. Med. 48:200. doi: 10.3892/ijmm.2021.5033, PMID: 34515322 PMC8448546

[ref24] Turner J. S. Kim W. Kalaidina E. Goss C. W. Rauseo A. M. Schmitz A. J.. (2021). SARS-CoV-2 infection induces long-lived bone marrow plasma cells in humans. Nature 595, 421–425. doi: 10.1038/s41586-021-03647-4, PMID: 34030176

[ref25] Vitiello L. Gatta L. Ilari S. Bonassi S. Cristina M. Ciatti F.. (2022). Long lasting cellular immune response induced by mRNA vaccination: implication for prevention strategies. Front. Immunol. 13:836495. doi: 10.3389/fimmu.2022.836495, PMID: 35359985 PMC8961295

[ref26] Wajnberg A. Amanat F. Firpo A. Altman D. R. Bailey M. J. Mansour M.. (2020). Robust neutralizing antibodies to SARS-CoV-2 infection persist for months. Science 370, 1227–1230. doi: 10.1126/science.abd7728, PMID: 33115920 PMC7810037

[ref27] Wang Z. Muecksch F. Schaefer-Babajew D. Finkin S. Viant C. Gaebler C.. (2021). Naturally enhanced neutralizing breadth against SARS-CoV-2 one year after infection. Nature 595, 426–431. doi: 10.1038/s41586-021-03696-9, PMID: 34126625 PMC8277577

[ref28] World Health Organization (2022). Weekly Epidemiological. Available at: https://www.who.int/publications/m/item/weekly-epidemiological-update-on-covid-19---1-june-2022 (Accessed June 1, 2022)

[ref29] Zollner A. Watschingera C. Röosslere A. Farcetf M. R. Pennerg A. Böohm V.. (2021). B and T cell response to SARS-CoV-2 vaccination in health care professionals with and without previous COVID-19. EBioMedicine 70:103539. doi: 10.1016/j.ebiom.2021.103539, PMID: 34391087 PMC8358275

[ref30] Zuo J. Dowell A. C. Pearce H. Verma K. Long H. M. Begum J.. (2021). Robust SARS-CoV-2–specific T cell immunity is maintained at 6 months following primary infection. Nat. Immunol. 22, 620–626. doi: 10.1038/s41590-021-00902-8, PMID: 33674800 PMC7610739

